# A pilot study to determine the effects of nasal co-phenylcaine on drug-induced sleep endoscopy

**DOI:** 10.1007/s00405-019-05519-0

**Published:** 2019-06-20

**Authors:** Alfonso Luca Pendolino, Ivor Kwame, Anne-Lise Poirrier, Maral J. Rouhani, Samit Unadkat, Giuliana Preti, Giancarlo Ottaviano, Peter J. Andrews, Bhik Kotecha

**Affiliations:** 1 Department of ENT, Royal National Throat, Nose and Ear Hospital, 330 Grays Inn Rd, Kings Cross, London, WC1X 8DA UK; 2 Department of Neurosciences, Otolaryngology Section, University of Padova, Padova, Italy; 3 ENT Department, University Hospital of Liege, Liege, Belgium; 4 Otolaryngology Section, Koelliker Hospital, Turin, Italy; 5 Ear Institute, UCL, London, UK

**Keywords:** Drug-induced sleep endoscopy, Obstructive sleep apnoea, Snoring, Nasal obstruction, Nasal decongestants, Nasal anaesthesia

## Abstract

**Purpose:**

The use of nasal decongestant and nasal anaesthesia is currently not recommended during drug-induced sleep endoscopy (DISE) according to the European position paper. The evaluation of the effects of nasal decongestant/anaesthesia on DISE has not been performed before and our aim is to perform a pilot study to determine whether nasal decongestants/anaesthesia affects DISE outcomes.

**Methods:**

27 patients undergoing DISE for OSA or for simple snoring were included. On each patient, DISE was performed twice, before and approximately 10 min after the administration of two puffs of co-phenylcaine nasal spray (lidocaine hydrochloride 5%, phenylephrine 0.5%, and benzalkonium chloride 0.01%) into each nostril. A nasal peak inspiratory flow was used for the objective assessment of nasal airway obstruction. During the first and the second DISE the loudness of the snoring was also recorded.

**Results:**

Change in DISE total grading after nasal spray administration was not statistically significant. For the same grading, changes in percentage of contribution to collapse were not statistically significant. Sex, AHI, BMI, tonsils grade, presence of rhinitis, turbinate hypertrophy, nasal septal deviation, or nasal peak inspiratory flow limitation had no influence on the effect of nasal spray. Co-phenylcaine did not significantly influence the loudness of snoring.

**Conclusions:**

Our pilot study supports the use of co-phenylcaine nasal spray during DISE and the positive effects of the nasal spray do not influence the grading outcome. Importantly, the decongestant enhances the nasal assessment during DISE and potentially aids in the diagnosis of nasal obstruction while the nasal anaesthetic component may be beneficial by reducing nasal discomfort during DISE and thereby helping to reduce the total dose of intravenous anaesthetic administered. However, further studies on a larger population are needed to confirm our results.

## Introduction

Obstructive sleep apnoea (OSA) is the most common sleep-disordered breathing (SDB) disease encountered with a prevalence of 3–17% in the adult population and is associated with an increasing rate of morbidity and mortality [1, 2]. Drug-induced sleep endoscopy (DISE) is a widespread procedure and is considered today as the most common diagnostic procedure for upper airway (UA) endoscopic evaluation for snoring and obstructive sleep apnoea (OSA).

Since its first description in 1991, DISE has gained increasing popularity, and evidence reports a good correlation between DISE findings and treatment outcomes [3, 4]. UA evaluation in awake OSA patients has limited usefulness because the patterns of UA collapse can change dramatically between awake and asleep, mostly due to differences in the muscle tone [5]. There remain controversies on how to perform sedation in DISE with an increasing need to reduce the amount of sedation infused and improve patient safety. There is an argument that local anaesthesia and nasal decongestant may reduce the inconvenience linked with scope insertion during DISE thus diminishing the total amount of sedation used. Given the known effects of nasal obstruction on SDB [6], nasal decongestant would also improve nasal evaluation during DISE by facilitating nasal endoscopic visibility and eliminating the rhinitis/congestion component of nasal blockage. On this regard, we have demonstrated that complete visualisation of both middle turbinates on anterior rhinoscopy predicts a good nasal airway and can be graded accordingly [7]. However, the European position papers do not recommend the use of nasal decongestants and nasal anaesthetics during DISE owing to the hypothesis that they may affect the grading score outcome [8, 9].

Our aim is to evaluate the effect of co-phenylcaine (lidocaine 5% and phenylephrine 0.5%) administered via nasal spray on 27 patients undergoing DISE for SDB disease and to evaluate if the application of co-phenylcaine could or not significantly affect the grading outcome and thereby influence the patient’s treatment plan.

## Materials and methods

### Patients

The present investigation was conducted in accordance with the 1996 Helsinki Declaration. Co-phenylcaine nasal spray during DISE has been intermittently used in our institute as common practice according to surgeons’ preference and, therefore, the current pilot study fell under local audit guidelines. Informed consent was obtained from each subject before starting any study-related procedure. Twenty-seven consecutive patients (19 men, 8 women) ranging from 20 to 75 years, with a mean age of 48.6 ± 11.9 years, undergoing DISE for OSA or for simple snoring at the Royal National Throat Nose and Ear Hospital were included. Demographic data, which included age, sex, Body Mass Index (BMI), main symptomatology (snoring vs OSA vs both) and previous surgery, were collected for all the patients. All patients were evaluated with home-based sleep studies (type III) before being included in the study, and the diagnosis of simple snoring or OSA was established according to the AHI calculated from the above-mentioned studies as follows: simple snoring, AHI < 5; mild OSA, 5 ≤ AHI < 15; moderate OSA, 15 ≤ AHI < 30; severe OSA, AHI ≥ 30. All subjects were asked to complete the Sino-Nasal Outcome Test (SNOT)-23, the Epworth Sleepiness Score (ESS), a Nasal Obstruction and Symptom Effectiveness scale (NOSE) questionnaire and a Visual Analogue Scale (VAS) for the symptom “nasal obstruction”.

### Clinical examination

A regular ENT examination in the sitting position was performed before DISE. Nasal Peak Inspiratory Flow (NPIF) was used for the objective assessment of nasal airway obstruction, and it was performed both in the sitting and supine position. A portable Youlten peak flow meter (Clement Clark International) was used for the NPIF measurements; two satisfactory maximal inspirations were obtained each time and the highest was then considered. A nasal endoscopy with a flexible fiberoptic endoscope was also performed prior to the DISE, to show the presence of a nasal septal deviation, inferior turbinate hypertrophy or presence of rhinitis (nasal congestion or rhinorrhea). The presence of an external alar valve collapse was also investigated.

### Drug-induced sleep endoscopy (DISE)

The DISE was performed in a silent operating room with the patient lying supine. The fiberoptic laryngoscope (Olympus ENF-GP, diameter 3.7 mm, Olympus Europe GmbH, Hamburg, Germany), connected to a high-resolution video system (Karl Storz Endovision TRICAM, Tuttlingen, Germany), was introduced through the nose to assess the UA. The DISE was carried out with the collaboration of an anaesthetist who was responsible for drugs infusion. Sedation was achieved using a combination of Propofol + Midazolam infused with a bolus technique. During the procedure, ECG and oxygen saturation were continuously monitored. On each patient, DISE was performed twice, before and approximately 10 min after the administration of two puffs of co-phenylcaine nasal spray (lidocaine hydrochloride 5% w/v phenylephrine 0.5% w/v benzalkonium chloride 0.01%) into each nostril with the patient remaining asleep on the table in the operative room during the interval between the two endoscopies. The findings noted were graded according to the Kotecha/Lechner grading system [10], and the contributions to the snoring of the nasopharynx, oropharynx and tongue base were recorded. In particular, in this grading system, in a grade 1 only a palatal flutter is evident; grade 2 is characterized by 100% contribution from the nasopharynx and no contribution from the oropharynx or tongue base; grade 3 correlates to multi-level collapse from velopharynx, oropharynx and tongue base only on inspiration, with different percentages of contribution for each level; grade 4 is similar to grade 3 but is present both on inspiration and expiration; grade 5 correlates to 100% contribution from the tongue base. Therefore, in all grade 3 and 4 the percentage of contribution in the UA collapse for each level must be specified. Grading was blindly confirmed by two different surgeons. During the first and the second DISE the loudness of the snoring was also recorded using a simple app for smartphone, with the phone positioned on the patient’s pillow at approximately 10 cm from his right ear.

### Statistical analysis

All data were collected prospectively. Quantitative variables were summarized using median and interquartile range (P25–P75) while qualitative variables were described with frequency and percentage. Since data were not normally distributed, non-parametric tests were used. Pre- and post-co-phenylcaine DISE outcomes were compared with Wilcoxon test for paired samples as primary endpoint. Odds ratios and 95% confident interval (CI95%) were calculated as secondary endpoints to evaluate the risk to change DISE outcome, loudness of snoring and management plan. Chosen factors were sex, AHI ≥ 15/h, BMI ≥ 30 kg/m^2^, tonsils grade ≥ 2, presence of rhinitis, turbinate hypertrophy, nasal septal deviation, NPIF limitation. Sex, AHI, BMI and enlarged tonsils were chosen because of their clinical relevance in OSA [11]. Rhinitis, turbinate hypertrophy, nasal septal deviation and NPIF limitation were chosen because of their known interference with nasal decongestant [12]. Statistical analysis was carried out using the free software *R* with *R* Commander [13]. Result were considered significant at the uncertainty level of 5% (*p* < 0.05).

## Results

Clinical and demographic data are summarized in Table 1. Among the 27 cases, 17 did not have a change in DISE outcome, eight showed a change in percentage of contribution to upper airway collapse and two patients showed a change in total DISE grading. Findings observed at the examination are reported in Table 2.

**Table 1:** Demographic and clinical data. Quantitative variables are summarized by median and interquartile range (P25–P75) while qualitative variables are described by frequency and percentage

	Subjects (*n* = 27)
Age, median [P25–P75], year	48 [41–55]
Sex, no (%)	
Female	8 (29.6%)
Male	19 (70.4%)
BMI, median [P25–P75], Kg/m^2^	27.5 [25.2–31.0]
Main complain, no (%)	
Snoring	9 (33.3%)
Snoring + OSA	18 (66.7%)
AHI, median [P25–P75], *n*/h	10.0 [4.1–20.2]
AHI, no (%)	
Simple snorers	8 (29.6%)
Mild OSA	10 (37%)
Moderate OSA	7 (26%)
Severe OSA	2 (7.4%)
ESS, median [P25–P75], *n*/24	12.0 [7.0–14.0]
Previous sleep surgery, no (%)	11 (40.7%)
NPIF, median [P25–P75], L/min	130.0 [87.5–155.0]
SNOT-23, median [P25–P75]	47.0 [33.5–57.0]
NOSE, median [P25–P75]	12.0 [7.5–15.5]
VAS (nasal obstruction/10)	5.0 [2.5–7.0]
Tonsil grade, no (%)	
0	7 (25.9%)
1	11 (40.7%)
2	7 (25.9%)
3	2 (7.4%)
4	0 (0.0%)
Friedman tongue position, no (%)	
1	8 (29.6%)
2	16 (59.3%)
3	3 (11.1%)
4	0 (0.0%)
Nasal septal deviation, no (%)	18 (66.7%)
Rhinitis, no (%)	21 (77.8%)
Inferior turbinate hypertrophy, no (%)	20 (74.1%)
External alar valve collapse, no (%)	0 (0%)

*BMI* Body Mass Index, *AHI* Apnea Hypopnea Index, *ESS* Epworth Sleepiness Scale, *NPIF* Nasal Peak Inspiratory Flow, *SNOT-23* Sino-Nasal Outcome Test-23, *NOSE* Nasal Obstruction and Septoplasty Effectiveness scale, *VAS* Visual Analogue Scale

**Table 2:** DISE outcomes before and after co-phenylcaine nasal spray

	Before co-phenylcaine	After co-phenylcaine	*p*-value
Total grading			
Median [P25–P75]	3 [3–3]	3 [3–3]	1.000
Mean ( ± sd)	3.4 ( ± 0.9)	3.4 ( ± 0.8)	
Contribution to collapse (%)			
Velopharynx, median [P25–P75]	31.3 [20.0–36.9]	25.0 [20.0–38.8]	0.2702
Oropharynx, median [P25–P75]	38.8 [30.6–50.0]	35.0 [20.0–53.8]	0.5261
Tongue base, median [P25–P75]	30.0 [20.0–35.0]	30.0 [20.0–38.8]	0.6089

**Fig. 1:**
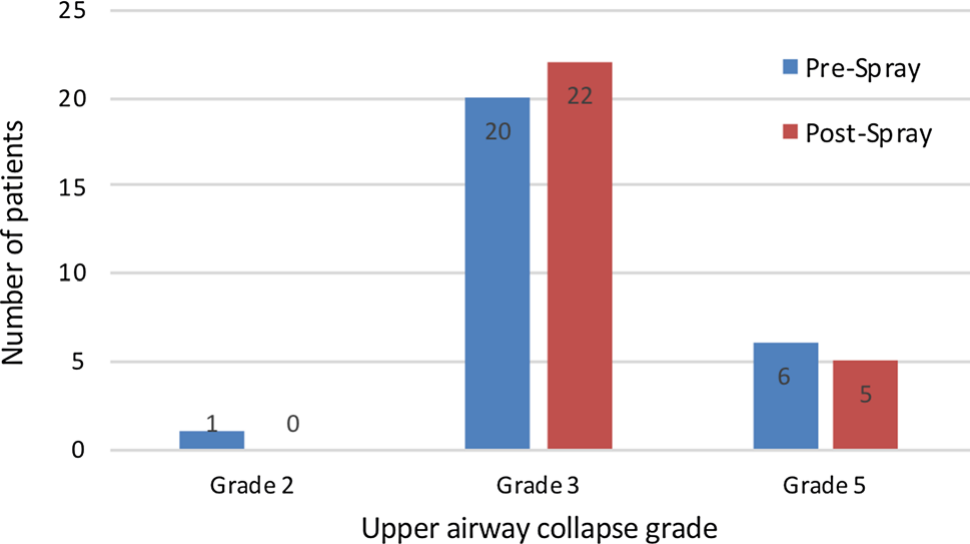
Upper airway collapse grade before and after spray administration

**Fig. 2:**
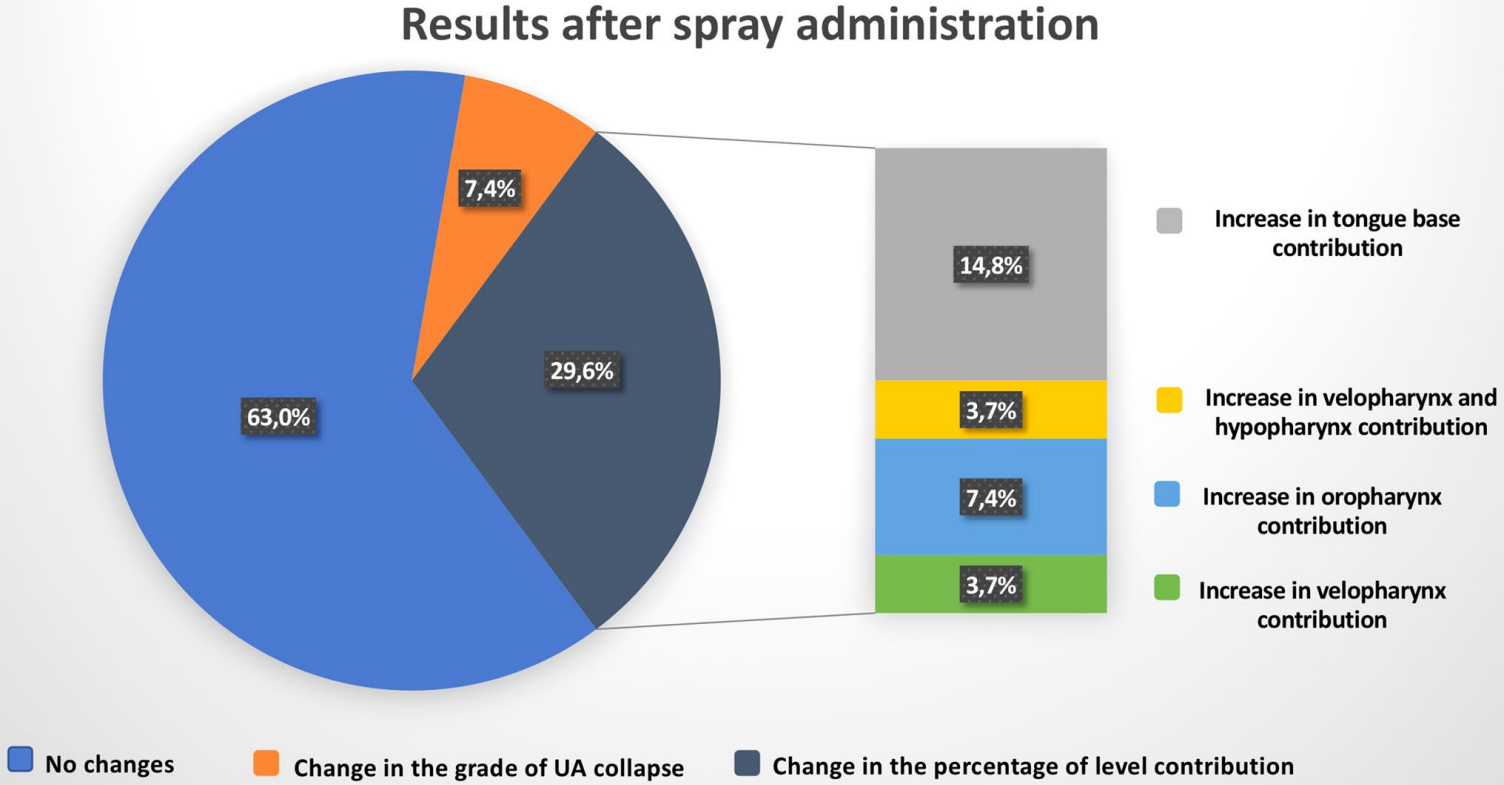
Results observed after spray administration

Considering UA collapse, the most frequent UA collapse grade recorded in our population was Grade 3, both before and after nasal spray application. Upon first performing DISE, 1 patient (3.7%) had grade 2 UA collapse, 20 patients (74.1%) had grade 3 and 6 patients (22.2%) had grade 5 UA collapse. After nasal spray administration 22 patients (81.5%) had a grade 3 and 5 patients (18.5%) a grade 5 of UA collapse. (Fig. 1) Analysing the findings obtained after spray administration, 17 patients (63%) showed no changes in terms of UA collapse grade or of percentage in level contribution (in case of a multi-level collapse). We observed a change in the percentage of level contribution (without a change in the grade) in only 8 patients (29.6%), all of them with a grade 3 UA collapse. Of these patients, one of them had an increase in velopharynx percentage contribution, two in the oropharynx, one in both the velopharynx and the oropharynx, and four showed an increase in the tongue base percentage contribution. (Fig. 2) However, change in DISE total grading after nasal spray administration was not statistically significant (*p* = 1.000 with effective Spearman pairing *r* = 0.841). Co-phenylcaine nasal spray did not change the total Kotecha/Lechner grading system with a statistical power (1 − *β*) of 0.92. For the same grading, changes in percentage of contribution to collapse were not statistically significant (*p* = 0.2702 for velopharynx, *p* = 0.5261 for oropharynx and *p* = 0.6089 for tongue base). Interestingly, in two patients (7.4%) we observed a change in the grade of UA collapse. (Fig. 2) In particular, one patient had first a grade of 5 and improved to a grade 3, and the other was initially grade 2 and then changed to grade 3 after spray administration.

Considering the loudness of snoring, we noticed an increase in loudness following nasal spray application in 13 patients (48%) (*p* = 0.0046). However only in six of them the increase in snoring was also associated with a change of UA collapse grade or in the percentage of level contribution.

We could not find any significant factor that could influence the effect of co-phenylcaine on DISE outcome, loudness of snoring or management plan. Nasal spray application did not affect the results of DISE independent of sex, AHI, BMI, tonsils grade, presence of rhinitis, turbinate hypertrophy, nasal septal deviation, or NPIF limitation.

## Discussion

Our results demonstrate that the use of co-phenylcaine does not significantly influence the DISE outcome and importantly does not change the patient’s management plan. Nasal spray application did not affect the results of DISE independent of the gender, AHI, BMI, tonsil grade, presence of rhinitis, turbinate hypertrophy, nasal septal deviation, or NPIF limitation.

According to the position papers on DISE, the use of local anaesthetic during the procedure is not recommended due to the possibility of having an influence on the tone of the pharyngeal muscles of the UA [8, 9]. The exact mechanism through which nasal local anaesthesia may interfere with palatal and pharyngeal muscles is not completely clear. Recent information suggests that the mechanism inducing arousal from sleep during airway occlusion in patients with OSA can be controlled by a complex evaluation made by the central nervous system of both the chemoreceptor stimulation (hypoxia and hypercapnia), the level of effort based on the level of ventilatory drive and/or the feedback from mechanoreceptors in the respiratory muscles or in the UA. Nevertheless, the contribution of UA mechanoreceptors to the arousal stimulus has not been clearly demonstrated. Topical nasal anaesthetic could increase apnoea duration by reducing the input from mechanoreceptors in the UA, even if results reported in the literature on this regard are conflicting. Basner and co-workers reported an increase in the time to arousal from non-rapid eye movement after application of 4% lidocaine to the oral and nasal mucosa [14]. Similarly, Berry et al. showed that topical UA anaesthesia increased the duration of obstruction apnoeas and the levels of inspiratory effort (oesophageal pressure deflection) prior to arousal from non-REM sleep [15]. Conversely, Redolfi et al. reported that topical lidocaine sprayed in the nasal and buccal cavities had no effects on respiratory-related evoked potentials [16]. Nasal negative pressure receptors may mediate activation of UA dilator muscles in the presence of nasal inspiratory negative pressure and/or airflow, and selective anaesthesia of the nasal mucosa is able to cause a decreased activation of the genioglossus and alae nasi muscles [17–19]. In addition, the effect of topical anaesthesia on UA receptors has also been investigated after topical oropharyngeal application of anaesthetic, but the results are contradictory [20–22]. Interestingly, Deegan and colleagues hypothesized that UA protective reflexes might not be important in patients with OSA and that these reflexes might already be significantly impaired in these patients, suggesting that oropharyngeal anaesthesia might have little or no additive effect in this category of patients [22].

Even in the case of nasal decongestant, the position papers do not advocate its use as it may interfere with the nasal resistance, thus influencing the airflow and the dynamics of the UA [8, 9]. Specifically, in patients with SBD disease, an increased nasal resistance results in UA closure in snorers [23] and OSA patients [24]. In addition, the volume of the pharyngeal mucosa plays an important role in controlling UA diameter, by maintaining pharyngeal patency, and topical application of phenylephrine in the UA resulted in a decrease in mucosal water content and a consequent increase in the pharyngeal cross-sectional area [25]. On this regard, Hutt et al. reported a significant reduction in SDB in patients with OSA after oropharyngeal and nasopharyngeal topical application of oxymetazoline [26]. It has also been proposed that the instillation of phenylephrine in the UA, by causing a reduction in the UA calibre, may reflexly activate some afferent fibres responding to changes in the size of the pharyngeal airway, finally leading to a further decrease in UA resistance [27–29]. Conversely, Wasicko et al. found no changes in UA muscle activity after pharyngeal and nasal plus pharyngeal instillation of phenylephrine [30] and, more recently, Clarenbach et al. concluded that the efficacy of nasal decongestion is not sufficient to provide a clinically substantial improvement of OSA, even if a reduction of the apnoea/hypopnoea index was observed [31].

In our department, co-phenylcaine is commonly administered prior to a nasal endoscopy to reduce the unpleasantness linked with the scope insertion, due to the high sensitivity of the nasal mucosa. Considering the advantages linked to the use of nasal anaesthesia and decongestant, as already mentioned, we decided to investigate if their use during DISE could modify the findings observed before their administration. In our study, co-phenylcaine nasal spray caused a modification in the DISE findings in 10 patients (37%). In particular, in 8 of them (29.6%) this modification consisted in a change in the percentage of level contribution, even if it was not so relevant to cause a plan modification. Furthermore, only in 2 patients (7.4%) this alteration implicated a change in the grade of UA collapse and a consequent modification of the surgical plan.

Contrary to our expectations, we observed in 13 patients (48%) an increase in snoring after nasal spray administration. In fact, it can be speculated that, as nasal decongestion increases nasal airway volume, patients should experience a higher nasal airflow, thus leading to an opening of UA and a consequent decrease of snoring. However, our findings are in line with previous studies which suggest that a reduction in nasal resistance during sleep may not correlate with snoring [32–34]. Nonetheless, we were unable to find a statistically significant relationship between spray administration and snoring.

The nose plays an important role in the development of SDB as nasal breathing is physiologically the preferential breathing route in wakefulness and in sleep [35]. Nasal obstruction in normal volunteers markedly increases the number of obstructive apnoeas and hypopneas during sleep [36–38]. In addition, patients with symptoms of rhinitis or with nasal congestion are at higher risk of developing snoring or OSA [39, 40] and a correlation has been found between total nasal resistance, AHI and oxygen desaturation [41]. Interestingly, in our study odds ratio calculation showed no influence of rhinitis, turbinate hypertrophy, nasal septal deviation or nasal obstruction (low NPIF) on the changes observed in DISE after spray application as well as the increase in the loudness of snoring. These results may suggest that improvement of nasal resistances, by means of nasal decongestants, may not influence UA collapse and snoring. A hypothesis that may explain our results is that oral breathing could be the preferred breathing route not only in subjects with a chronic nasal obstruction [36–40] but also in chronic apnoeics and snorers without a nasal obstruction [42, 43]. Thus, an improvement in nasal resistances may be irrelevant in improving UA collapse or decreasing snoring, at least in the short term.

A limitation of our study was the lack of a tool to assess the depth of sedation and anaesthesia, [e.g. bispectral (BIS) index or cerebral state index (CSI)]. It can be argued that, due to the absence of this tool, we cannot be sure if the changes observed 10 min after co-phenylcaine application were a consequence of the spray administered or of the deepening of the sedation. However, our experience is based on thousands of DISE procedures performed each year and sedation is administered by high-skilled anaesthetists in this procedure. In addition, as we used a spray made by a combination of lidocaine and phenylephrine, we were not able to distinguish if the results observed were caused by one of the two drugs or by a combination of both.

A further limitation of our study was that a post decongestant NPIF was not performed as this would have allowed us to potentially determine the NPIF diagnostic percentage change and help differentiate between congestable and structural causes for nasal obstruction [44]. Overall, there is a need to improve the rhinological assessment component during DISE. However, our pilot study only recruited a small number of patients and although the evidence is supportive there is a need for a larger study to fully evaluate the benefits of using co-phenylcaine during DISE and the need to expand on the rhinological assessment of SDB.

## Conclusions

Our pilot study supports the use of co-phenylcaine nasal spray during DISE. The advantages of using a nasal decongestant include improving nasal endoscopic visibility through the elimination of nasal congestion as well as aiding in the diagnosis of congestible versus structural causes of nasal congestion. The anaesthetic component could also lower the total dose of intravenous anaesthetic administered during the procedure by reducing patient discomfort.
